# Proteomic characterization of extracellular vesicles released by third stage larvae of the zoonotic parasite *Anisakis pegreffii* (Nematoda: Anisakidae)

**DOI:** 10.3389/fcimb.2023.1079991

**Published:** 2023-03-15

**Authors:** Marialetizia Palomba, Aurelia Rughetti, Giuseppina Mignogna, Tiziana Castrignanò, Hassan Rahimi, Laura Masuelli, Chiara Napoletano, Valentina Pinna, Alessandra Giorgi, Mario Santoro, Maria Eugenia Schininà, Bruno Maras, Simonetta Mattiucci

**Affiliations:** ^1^Department of Ecological and Biological Sciences, University of Tuscia, Viterbo, Italy; ^2^Department of Experimental Medicine, Sapienza University of Rome, Rome, Italy; ^3^Department of Biochemistry Science, Sapienza University of Rome, Rome, Italy; ^4^Department of Integrative Marine Ecology, Stazione Zoologica Anton Dohrn, Naples, Italy; ^5^Department of Public Health and Infectious Diseases, Section of Parasitology, Sapienza University of Rome, Rome, Italy

**Keywords:** *Anisakis pegreffii*, zoonotic parasite, third stage larvae, extracellular vesicles, proteomics, heat shock proteins, metalloproteases, allergenic proteins

## Abstract

**Introduction:**

*Anisakis pegreffii* is a sibling species within the *A. simplex* (s.l.) complex requiring marine homeothermic (mainly cetaceans) and heterothermic (crustaceans, fish, and cephalopods) organisms to complete its life cycle. It is also a zoonotic species, able to accidentally infect humans (anisakiasis). To investigate the molecular signals involved in this host-parasite interaction and pathogenesis, the proteomic composition of the extracellular vesicles (EVs) released by the third-stage larvae (L3) of *A. pegreffii*, was characterized.

**Methods:**

Genetically identified L3 of *A. pegreffii* were maintained for 24 h at 37°C and EVs were isolated by serial centrifugation and ultracentrifugation of culture media. Proteomic analysis was performed by Shotgun Analysis.

**Results and discussion:**

EVs showed spherical shaped structure (size 65-295 nm). Proteomic results were blasted against the *A. pegreffii* specific transcriptomic database, and 153 unique proteins were identified. Gene Ontology and Kyoto Encyclopedia of Genes and Genomes analysis predicted several proteins belonging to distinct metabolic pathways. The similarity search employing selected parasitic nematodes database revealed that proteins associated with *A. pegreffii* EVs might be involved in parasite survival and adaptation, as well as in pathogenic processes. Further, a possible link between the *A. pegreffii* EVs proteins *versus* those of human and cetaceans’ hosts, were predicted by using HPIDB database. The results, herein described, expand knowledge concerning the proteins possibly implied in the host-parasite interactions between this parasite and its natural and accidental hosts.

## 1 Introduction

*Anisakis pegreffii* is a sibling species of the *A. simplex* (s.l.) species complex (Mattiucci et al., 2014). It has a complex life cycle involving mainly cetaceans of the superfamily Delphinoidea as definitive hosts, planktonic or semi-planktonic crustaceans as first intermediate hosts, while pelagic and mesopelagic fish and cephalopods act as intermediate/paratenic ones (Mattiucci et al., 2018a; Mattiucci et al., 2021; Cipriani et al., 2022). Thus, it requires homeothermic hosts, in which the adult parasites live and sexually reproduce, and heterothermic hosts in which the third stage larvae (L3) spread. These parasites do not have a high pathogenic effect on their natural hosts (Santoro et al., 2018; Dezfuli et al., 2021) as likely the result of a long co-evolutionary history which would have led to reciprocal adaptation phenomena (Mattiucci and Nascetti, 2008). L3 infects edible parts of fish or cephalopods (Mattiucci et al., 2018a; Cipriani et al., 2021; Palomba et al., 2021a). In humans, raw and/or undercooked infected fish/cephalopods can provoke a zoonotic disease, known as anisakiasis. The species *Anisakis pegreffii*, as well as *A. simplex* (s.s.), are known as etiological agents of gastric, intestinal, gastroallergic, and ectopic anisakiasis in several countries (Mattiucci et al., 2018a; Mattiucci et al., 2021; Sugiyama et al., 2022).

In the last years, several studies have been carried out to investigate the biological mechanisms and molecular signals involved in the human accidental host infection and pathogenesis of *Anisakis* spp. (Baird et al., 2016; Bušelić et al., 2018; Cavallero et al., 2018; Llorens et al., 2018; Harbar et al., 2019; Palomba et al., 2019; Łopieńska-Biernat et al., 2020; Marzano et al., 2020; Stryiński et al., 2020; Trumbić et al., 2021; Kochanowski et al., 2022; Polak et al., 2022). Nevertheless, studies focused to investigate molecules shaping the adaptation of these parasites to their natural hosts are rather limited (Mehrdana and Buchmann, 2017; Palomba et al., 2020; Trumbić et al., 2021; Harbar et al., 2022). Recent studies have revealed that extracellular vesicles (EVs) represent a new paradigm in the “cross-talk” between parasites and their hosts, playing a crucial role in pathogenesis’s mechanisms including the parasite’s capacity for immune evasion (Marcilla et al., 2012; Barteneva et al., 2013; Marcilla et al., 2014; Coakley et al., 2015; Sánchez-López et al., 2021; White et al., 2022a). In addition, helminth-derived EVs are recently proposed as key players in helminth-microbiota crosstalk (Rooney et al., 2022). EVs are membrane-enclosed nanoparticles released by almost each cell type. They can be formed by outward budding of the plasma membrane or generated by the reshuffling of intracellular membranes (multivesicular bodies, MVBs) and, subsequently, released outside (Yáñez-Mó et al., 2015; Drurey and Maizels, 2021). Depending on size and biogenesis, EVs have been categorized in exosomes (40-100 nm) originating from MVBs, and microvesicles (100 nm to 1 μm) originating from plasma membranes (Evans-Osses et al., 2015). EVs can constitute for the parasites’ species an alternative export mechanism to release outside the proteins possibly involved in pathogenic and immunomodulating mechanisms (Colombo et al., 2014; Tritten and Geary, 2018; Sánchez-López et al., 2021). So far, the proteomic composition of EVs have been reported in *Anisakis* spp. (Boysen et al., 2020), while miRNAs have been studied and recorded in *A. pegreffii* (Cavallero et al., 2022a).

This study aims to investigate the proteomic repertoire associated with EVs of *A. pegreffii* L3 maintained *in vitro* at the temperature of 37°C and characterize the parasite proteins which might be involved in the interaction with definitive (marine mammals) and accidental (humans) hosts.

## 2 Materials and methods

### 2.1 *Anisakis* L3 sampling and *in vitro* culture

*Anisakis* larvae were extracted using scissors and tweezers from the body cavity of three female of silver scabbardfish (*Lepidopus caudatus*) (Mean total length ± SD, 128,6 cm ± 47,25) caught approximately 12 h before from the Adriatic Sea (off San Benedetto del Tronto coast), a fishing area with a known high prevalence of *Anisakis* infection (Cipriani et al., 2018). After their removal, the larvae were checked for their integrity under a dissecting microscope and the third larval stage was assigned by morphological criteria to Type I larvae (*sensu* Berland, 1961). Their vitality was evaluated based on their spontaneous movements. Alive and not disrupted larvae were washed in a sterile 1X phosphate-buffered saline solution (PBS, Sigma, St Louis, MO) three times (30 worms/mL) for 1 min each, treated for 1 min with 4% acetic acid (Carlo Erba, Cornaredo, Italy) to inhibit bacterial contamination and rewashed in the sterile PBS for 1 min. Then, the larvae were cultured in filtered sterile PBS (30 larvae/mL/well) with 1% pen-strep in 12 well plates for 24 h, in humified atmosphere at 37°C, 5% CO_2_. Three biological replicates were performed.

### 2.2 Molecular identification of *Anisakis* L3

A representative subsample of 100 *Anisakis* larvae taken among those cultured, was used for molecular identification. Total genomic DNA from each larva was extracted using the Quick-gDNA Miniprep Kit (ZYMO RESEARCH) following the procedure reported in Irigoitia et al., 2021. The mitochondrial cytochrome *c* oxidase 2 (mtDNA *cox*2) gene locus was amplified using the primers 211F (forward; 5′-TTTTCTAGTTATATAGATTGRTTYAT-3′) and 210R (reverse; 5′-CACCAACTCTTAAAATTATC-3′) (Valentini et al., 2006; Mattiucci et al., 2014). The successful PCR products were purified, and Sanger sequenced through an Automated Capillary Electrophoresis Sequencer 3730 DNA Analyzer (Applied Biosystems), using the BigDye^®^ Terminator v3.1 Cycle Sequencing Kit (Life Technologies). Additionally, a direct genotyping determination of the nuclear metallopeptidase 10 gene locus (*nas*10 nDNA) was performed by the amplification-refractory mutations system (ARMS) PCR assay at *nas*10 nDNA by the combined use of OUT-F1 (forward; 5’-TATGGCAAATATTATTATCGTA-3’), OUT-R1 (reverse; 5’-TATTTCCGACAGCAAACAA-3’), INN-F1 (forward; 5’-GCATTGTACACTTCGTATATT-3’), INN-R1 (reverse; 5’-ATTTCTYCAGCAATCGTAAG-3’), following the procedures reported in Palomba et al. (2021b). PCR products were separated by electrophoresis using agarose gel (1.5%) stained with GelRed. The distinct banding patterns were detected using ultraviolet transillumination.

### 2.3 Isolation of extracellular vesicles

Following the incubation period (24 h, at 37°C, 5% CO_2_), L3 were manually removed, and their viability was checked under a stereomicroscope (Leica M205, FCA). The larval culture supernatant was collected, and a protease inhibitor (12,5X/mL) (cOmplete, EDTA-free, Roche) was added, following the standard protocol. Then, EVs were immediately isolated as previously described (Battisti et al., 2017). Briefly, the supernatant was centrifuged twice (4000 rpm, 30 min, 2 times). The cleared supernatant underwent serial ultracentrifugation steps (10,000 *g*/1 h and 100,000 *g*/80 min). The pellet was then washed in PBS (100,000 *g*/80 min). Ultracentrifugation was performed employing Swing 55 rotor and Beckman ultracentrifuge. Finally, the pellet was collected and resuspended in 50 μl PBS and stored at -80°C. Protein concentration, Nanoparticle Tracking analysis (NTA) and proteomic analysis were performed within two weeks from isolation. For Transmission Electron Microscopy, freshly isolated EVs were used. Protein concentration was tested by Bradford assay; purity of the EVs was evaluated as ratio between number of particle and μg of protein (P/μg) (Webber and Clayton, 2013; Théry et al., 2018).

### 2.4 Nanoparticles tracking analysis of EVs

Size determination of the isolated EVs was performed by nanoparticles tracking analysis (NTA) (Dragovic et al., 2011). EVs were thawed on ice and diluted 1:500 in filtered PBS (20 nm filter) and vortexed to achieve the optimal number of EVs/mL ratio. Three videos (30 s each) were recorded for each sample loading, employing the NanoSight NS300 instrument (Malvern Instruments Ltd, Malvern, UK). Measurements were performed employing the NTA 2.3 analytical software. Results were shown as the average of the three recordings.

### 2.5 Transmission electron microscopy of EVs

Transmission Electron Microscopy (TEM) of EVs was performed according to Borrelli et al. (2018). Briefly, freshly isolated EVs were fixed in 2% paraformaldehyde and adsorbed on formvar-carbon-coated copper grids. The grids were then incubated in 1% glutaraldehyde for 5 min, washed with deionized water eight times, and then negatively stained with 2% uranyl oxalate (pH 7.0) for 5 min and methyl cellulose/uranyl for 10 min at 4°C. Excess methyl cellulose/uranyl was blotted off, and the grids were air dried and observed with a TEM (Philips Morgagni 268D) at an accelerating voltage of 80 kV within 48 h from staining. Digital images were taken with Mega View imaging software.

### 2.6 Proteomic analysis of EVs

Protein fraction was extracted from the EV preparation (Abramowicz et al., 2018). Briefly, the samples were mixed with acetonitrile to the final concentration of 50% (v/v), and after 45 min of incubation at RT with occasional mixing cycles, acetonitrile was evaporated using a centrifugal vacuum concentrator. Protein concentration was determined by Bradford assay (Biorad). A shotgun proteomic strategy was employed on the protein content of L3 *A. pegreffii* EVs. Briefly, approximately 7 μg of the sample was mixed with SDS and DTT, boiled, cooled to room temperature, and then alkylated with iodoacetamide in the dark for 30 min. Proteolysis was carried out in an S-Trap filter (ProtiFi; Huntington, NY) following the manufacturer’s procedure. Phosphoric acid (1.2% final concentration) and binding buffer (six volumes) were added. After gentle mixing, the protein solution was loaded to the S-Trap filter, spun at 2000 rpm, and the flow-through was collected and reloaded onto the filter. This step was repeated three times, followed by three times washing with binding buffer. Digestion buffer containing trypsin at 1:10 (w:w) was added into the filter and proteolysis was carried out. The final proteolytic peptide mixture was pooled, lyophilized, resuspended in 0.2% formic acid, and then split into three equal technical replicates, which were then analysed by liquid chromatography-mass spectrometry (LC-MS/MS) using LTQ Orbitrap XL (ThermoScientific, Waltha, MA, USA) coupled to a nanoHPLC system (nanoEasy II, ThermoScientific, Waltha, MA, USA). The three samples were loaded, concentrated, and desalted on a C18 Easy-Column (L = 2 cm, ID = 100 μm; cat. no. 03-052-619, ThermoScientific SC001). Fractionation online with the nanospray ESI source was then achieved on a C18 reverse-phase capillary column (L = 20 cm, ID = 7.5 μm; cat. no. NS-AC-12, NanoSeparations, Niewkoop, Netherlands) at a flow rate of 250 nl/min in a gradient from 5% to 95% of eluent solvent B (eluent B: 0.2% formic acid in 95% acetonitrile; eluent A: 0.2% formic acid and 2% acetonitrile in ultrapure water) over 285 min. The MS/MS acquisition method was set up in a data-dependent acquisition mode, with a full scan ranging from 400 to 1800 m/z range, followed by fragmentation in CID modality of the top 10 ions (MS/MS scan) selected based on intensity and charge state (+2, +3 charges). In the selection, an exclusion time of 40 seconds was applied.

### 2.7 Bioinformatics analysis

The EVs protein content was profiled through the quantitative proteomics software package MaxQuant (Max Planck Institute of Biochemistry, Martinsried, DE) (Tyanova et al., 2016), employing the Andromeda algorithm against the query database, its reverse decoy database, and a database of common contaminant proteins integrated into the MaxQuant package v. 1.6.0.16. In particular, a protein identity searching process was carried out on the LC-MS/MS spectra, collected from the three replicates, against a customized database achieved by the *de novo* transcriptome assembly of *A. pegreffii* L3 (97,480 peptide sequences) (Palomba et al., 2022). The following search parameters were used: trypsin as proteolytic enzyme; 2 as a maximum allowed missed cleavages; carbamidomethyl cysteine as fixed modification; oxidation of methionine and pyroglutamic acid at the peptide N-terminus as variable modifications; 7 as minimum peptide length considered in protein identification; 1% FDR both for peptide spectrum matching and for protein identification. The minimum number of peptides for protein identification was set to 4, with at least 3 unique peptides. Alignment between contiguous HPLC runs was activated. The validation of protein identification was based on the q-value. All further identification and quantification parameters were set as default. Only proteins that were identified by MS/MS analysis in all three replicates were accepted in the final protein list. To assess whether certain classes of proteins were enriched in EV proteome, the gene ontology (GO) analysis and the Kyoto Encyclopedia of Genes and Genomes (KEGG) pathways analysis for cellular components, molecular functions, and biological processes were carried out using eggNOG-mapper v. 2 (Cantalapiedra et al., 2021) and OMA browser (Altenhoff et al., 2021). InterPro protein family classification and enzyme identification were performed using OmicsBox with the Blast2GO algorithm (Götz et al., 2008). ClusterProfler and AnnotationHub74 were subsequently employed to obtain the enrichment analysis of proteins clusters. The predicted EV proteins were blasted against the known and available EV-associated proteins of nematodes, i.e., *Anisakis* spp. (Boysen et al., 2020), *Ascaris suum* (Hansen et al., 2019), *Nippostrongylus brasiliensis* (Eichenberger et al., 2018a), *Brugia malayi* (Harischandra et al., 2018) and *Trichuris muris* (Eichenberger et al., 2018b). The predicted EV proteins were blasted against seven databases. In detail, proteases and protease inhibitors, essential proteins for life, and potential allergic proteins were identified using the BLASTp search against MEROPS (The peptidase database) (Rawlings et al., 2011), DEG (Database of Essential Genes) (Luo et al., 2021) and FARRP (Food Allergy Research and Resource Program) databases, respectively. Potential allergens were also confirmed by the AllerCatPro 2.0 server (Maurer-Stroh et al., 2019). Putative pathogenicity-related proteins were detected using a BLASTp search against the protVirDB (Database of Protozoan Virulent Proteins), VICTORS (Virulence Factors database), and VFDB (Virulence Factors of Pathogenic Bacteria) databases. Host-parasite interactions were predicted using the HPIDB 3.0 (Kumar and Nanduri, 2010; Ammari et al., 2016), which was run with the default setting using EVs searched against human (*Homo sapiens*, UniProt proteome ID: UP000005640; # of entries 79,052) and bottlenose dolphin (*Tursiops truncatus*, UniProt proteome ID: UP000245320; # of entries 45,130) proteomes. Analyses were performed on the high-performance computing platforms provided by ELIXIR-IT HPC@CINECA (Castrignanò et al., 2020).

## 3 Results

### 3.1 Molecular identification of *A. pegreffii* L3

The BLAST analysis of the 100 sequences of *Anisakis* obtained at the mtDNA *cox2* gene locus (~600 bp) retrieved a percentage of identity of 99-100% with the sequences of *A. pegreffii* previously deposited (KY565564-KY565562). Additionally, the ARMS-PCR analysis enabled us to genotype the same individuals belonging to the species *A. pegreffii*. Briefly, the use of the *nas10* primers generated a specific PCR product of 117 bp, amplifying the C-allele (Palomba et al., 2021b).

### 3.2 *Anisakis pegreffii* L3 releases extracellular vesicles

**Figure 1:**
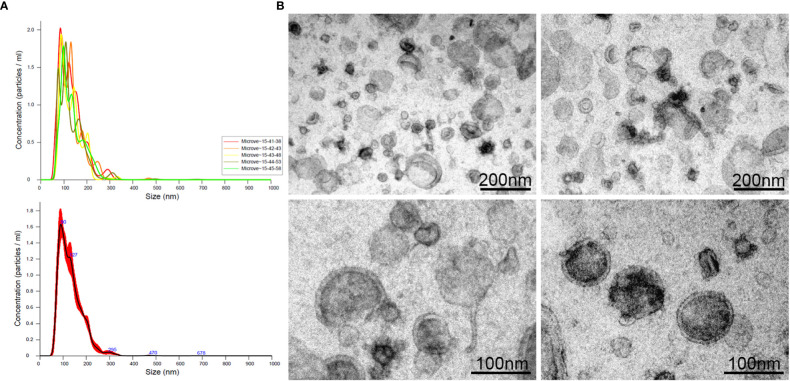
Extracellular vesicles (range: 40 – 450 nm) released in culture medium by L3 of *A. pegreffii* after 24h incubation, analysed by NTA **(A)** and visualized by TEM **(B)**. The scale bar is indicated in the figure.

Purified EVs, released during the 24h culture of L3 (L3-EVs) in PBS, were characterized by Nanoparticles Tracking Analysis (NTA). Results indicated that L3-EVs had an estimated vesicle size of 65-295 nm and peaked at a mean diameter of 132,3 ± 0.7 nm, which had the prototypical size characteristic of both microvesicles and exosomes (**Figure 1A**). The concentration was 1,54 x 10^11^ particles/mL, corresponding 5 x 10^9^ particles/worm and a protein content of 0,104 μg/mL. The P/μg was 1.48 x 10^12^. The morphology of the L3-EVs, investigated by TEM, showed that L3-EVs displayed a typical rounded-shaped structure, with lipid bilayer-bound membrane structures, approximately 80-240 nm in diameter (**Figure 1B**), fully in agreement with the NTA measurements.

### 3.3 Protein repertoire associate with *A. pegreffii* L3-EVs

The characterization of the protein landscape of the L3-EVs, obtained starting from the *de novo* transcriptomic data available for *A. pegreffii* (Palomba et al., 2022) inferred from the analysis of the so far transcriptomes published, allowed. to detect a total of 1083 transcripts. They matched with sequences determined by MS/MS spectra, permitting to identify 153 protein groups. Of those, 5 were unidentified proteins. With respect to the transcriptome used (i.e., 97480 contigs), the percentage of identified proteins was 0.2%. The results of the protein identification search are summarized in the **Supplementary File 1**.

### 3.4 Gene ontology annotation and Kyoto Encyclopedia of genes and genomes pathway identification

**Figure 2:**
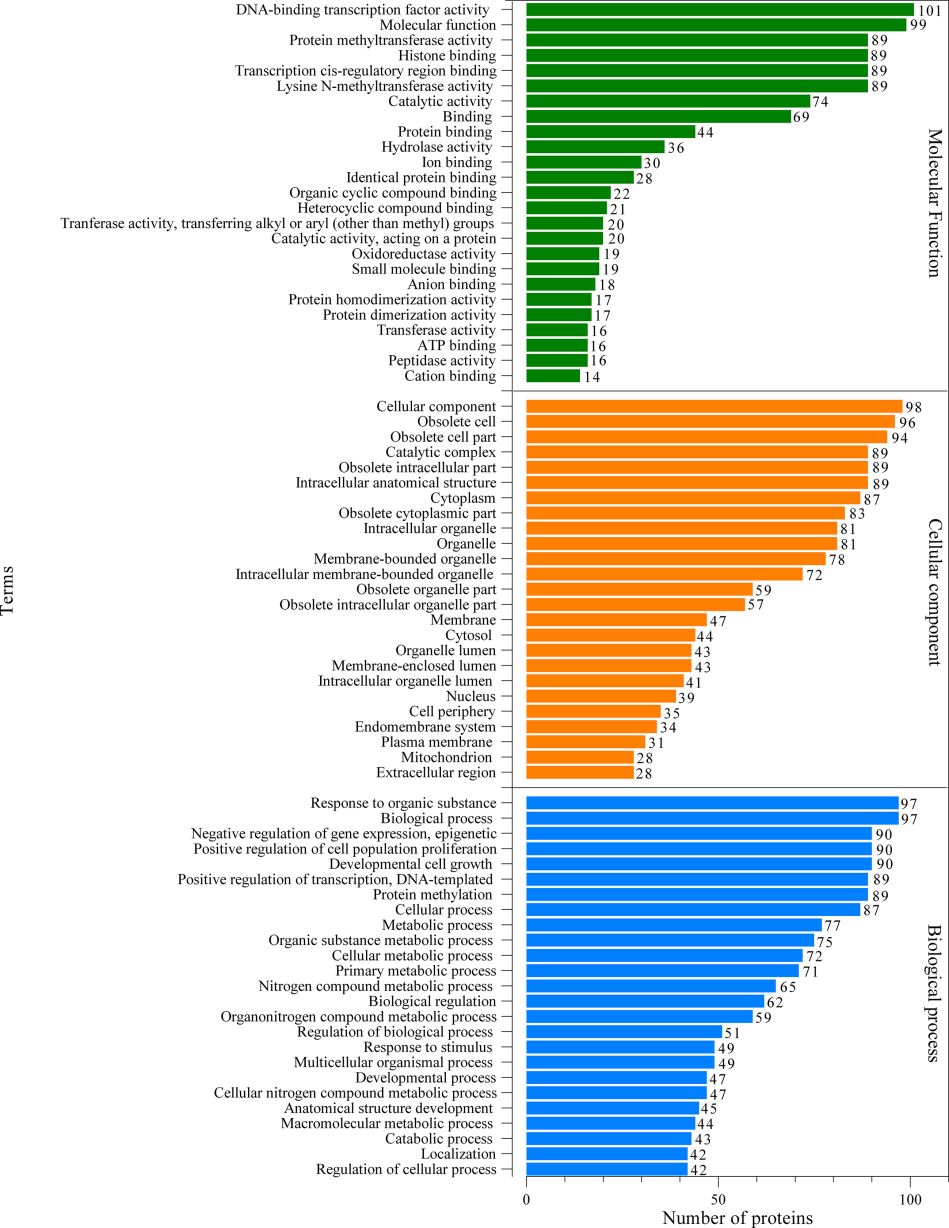
Gene ontology (GO) analysis of the predicted EVs proteins. Green, orange and blue bars represent molecular function, cellular component and biological process, respectively.

**Figure 3:**
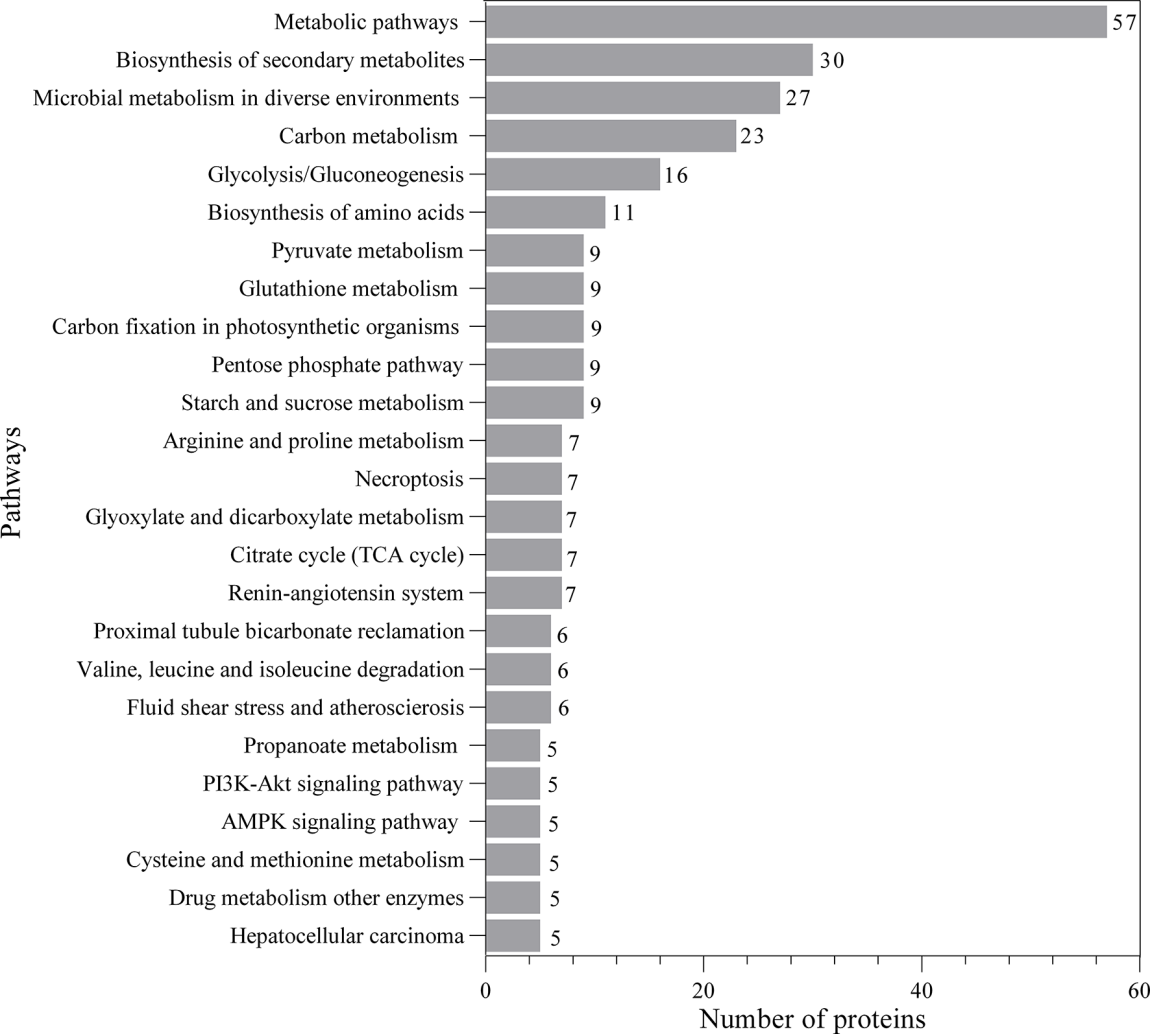
Kyoto Encyclopedia of Genes and Genomes (KEGG) analysis of predicted EVs proteins.

The proteomic components of L3-EVs were classified by GO annotation according to putative molecular function, biological process, and cellular compartments (**Figure 2**). Around 95% of the proteins were annotated with GO terms. In the molecular function category, there was a high prevalence of “DNA-binding transcription factor activity” (101 proteins); in the cellular component category the most abundant GO term was “cellular component organization” (98 proteins); finally, in the biological process category, the most frequent GO term was “response to organic substance” (97 proteins). A strong enrichment for 26 terms of the biological process category was found (**Supplementary Figure 1**). In the molecular category, a strong enrichment was found only for two terms “aminopeptidase activity, carboxypeptidase activity, metalloexopeptidase activity, aldehyde dehydrogenase (NAD+) activity” (**Supplementary Figure 1**). While no enrichment terms were found for the “cellular component” category. The KEGG analysis revealed the presence of proteins involved in various pathways (25 pathways) (**Figure 3**). The most frequent was the “metabolic pathway” (57 proteins), followed by “biosynthesis of secondary metabolites” (30 proteins) and “microbial metabolism in diverse environments” (27 proteins). A strong enrichment was observed for 22 pathways (**Supplementary Figure 2**).

### 3.5 Protein family and enzyme classification

**Figure 4:**
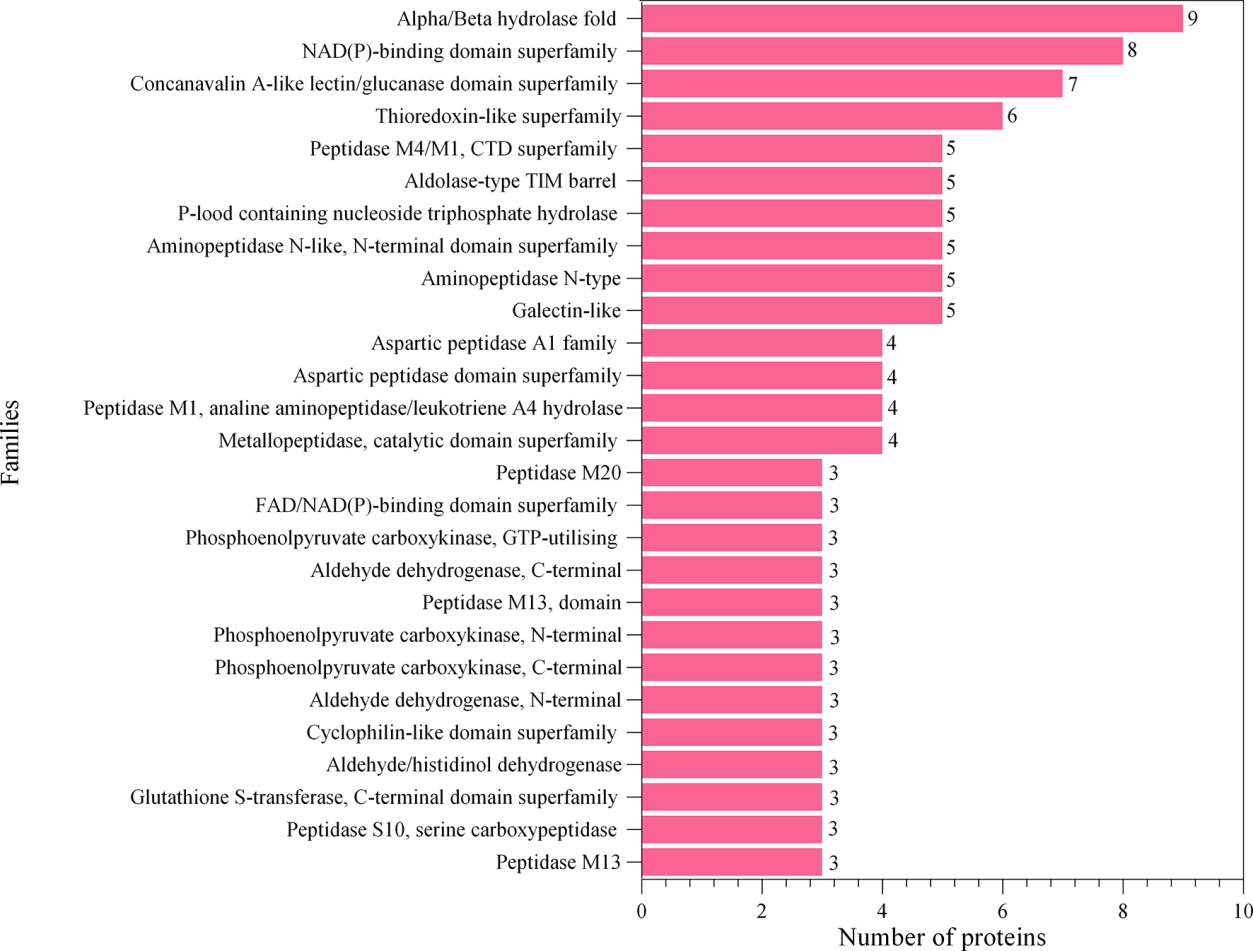
Families’ identification analysis of predicted EVs proteins.

**Figure 5:**
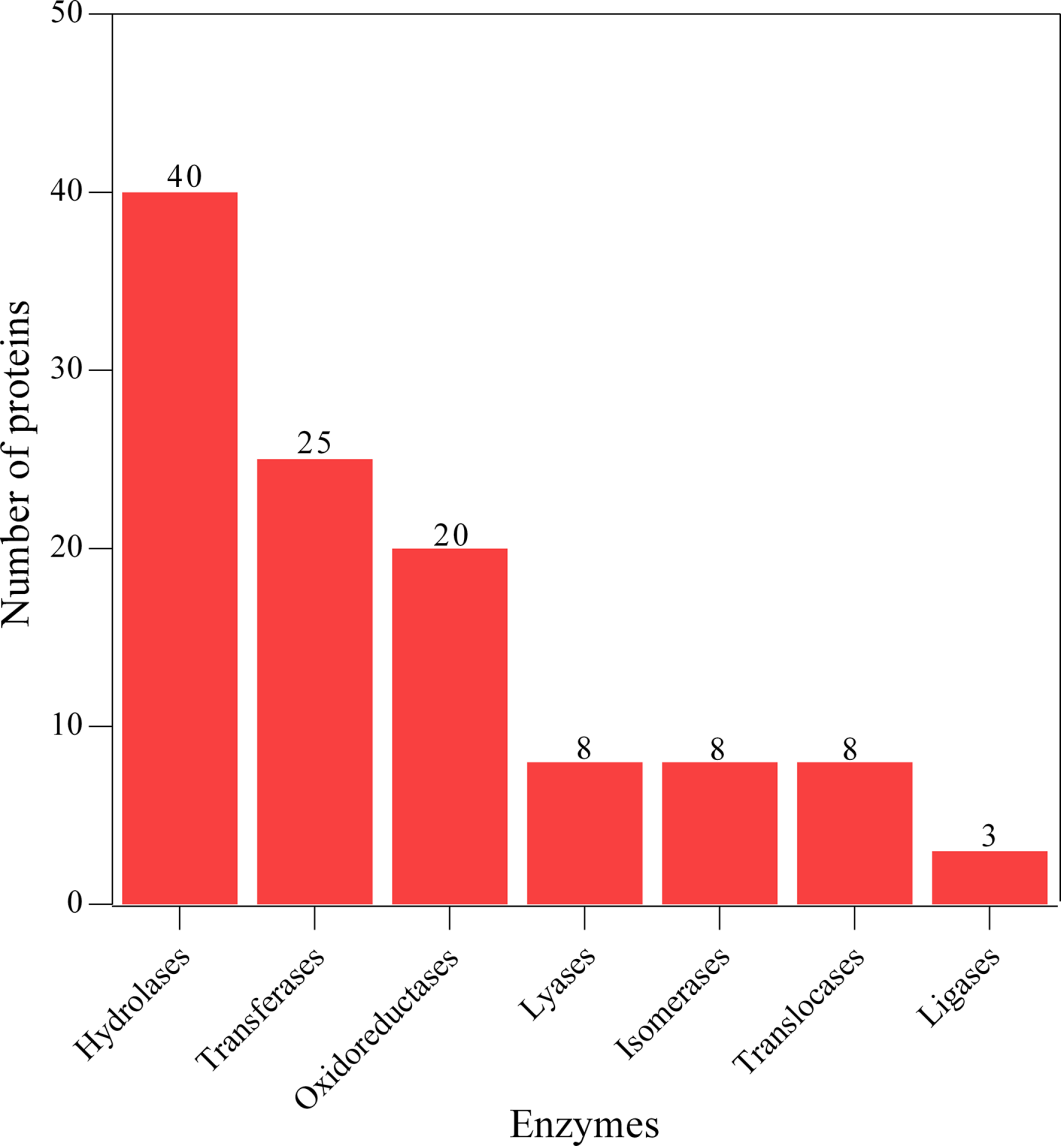
Enzyme class identification analysis of predicted EVs proteins.

The proteins associated with L3-EVs appeared to belong to distinct families. A total of 224 protein families were detected. Most of them (196 families) were represented by only two and one protein (37 and 159 families, respectively). The most represented are the alpha/beta hydrolase fold family (9 proteins), NAD(P)-binding domain superfamily (8 proteins), concanavalin A-like lectin/glucanase domain superfamily (7 proteins) and thioredoxin-like superfamily (6 proteins) (**Figure 4**). The most common type of proteins in L3-EVs are enzymes (73,2%). The most abundant class is represented by hydrolases (40), transferases (25), oxidoreductases (20), lyases (8), isomerases (8), translocases (8) and ligases (3) (**Figure 5**). In particular, the hydrolases class is represented by 3 glycosylases and enzymes acting on peptide bonds (peptidases, 27), ester bonds (4), acid anhydrides (3), ether bonds irata (1), hydrolase (1), and carbon-nitrogen bonds (1).

### 3.6 *Anisakis pegreffii* L3-EVs share protein repertoire with EVs of other nematode parasites

The protein repertoire associated with L3-EVs, when compared with that previously obtained from unidentified L3 of *Anisakis* spp. by Boysen et al. (2020), revealed a total of 11 proteins shared by the two data sets, showing a blast similarity ranging from 32.0 up to 100% (**Table 1**). In particular, they were: the chloride intracellular channel exc-4, the actin 2, the pepsin-I3 domain-containing protein, the p-type domain-containing protein, glutamate dehydrogenase (NAD(P) (+)), heat shock protein 70, 14-3-3 zeta, ras-related protein Rab-11B and three unnamed proteins (**Table 1**). The three unnamed proteins showed a blast similarity of 96.58%, 95.41% and 78.00% with two histidine acid phosphatases and prostatic acid phosphatase, respectively (**Table 1**).

**Table 1:** Top *A. pegreffii* L3-EVs proteins identified in the EVs of unidentified L3 of *Anisakis*.

EVs from *A. pegreffii*	EVs from *Anisakis* spp.		Blast Similarity (%)
Protein Name	UniProt Accession No.	Protein Name	
Chloride intracellular channel exc-4	A0A0M3K9P2	CBN-exc4	100
Actin-2	A0A0M3J0M4	Actin	100
Pepsin-I3 domain-containing protein	A0A0M3JAH0	Pepsin inhibitor	99.28
P-type domain-containing protein	A0A0M3KCN6	Maltase glucoamylase	98.66
Unnamed protein product	A0A0M3KA60	Histidine acid phosphatase	96.58
Unnamed protein product	A0A0M3J727	Histidine acid phosphatase	95.41
Glutamate dehydrogenase (NAD(P)(+))	A0A0M3K4H2	Glutamate dehydrogenase	93.28
Heat shock protein 70	A0A0M3K9V2	Heat shock protein 70	91.68
Unnamed protein product	A0A0M3JAF9	Prostatic acid phosphatase	78.00
14-3-3 zeta	A0A0M3K8U5	14-3-3 zeta	67.17
Ras-related protein Rab-11B	A0A0M3KFX3	Ras like protein 3	32.95

The carried proteins of L3-EVs, when compared with those available for other ascaridoid nematodes, i.e. *A. suum*, *N. brasiliensis B. malayi* and *T. muris* revealed that top ten proteins showed a blast similarity ranging from 93.8 up to 100% (**Table 2**). In particular, the actin 2, galectin, adenylate kinase isoenzyme 1, and the PPlase cyclophilin-type domain-containing protein were also detected in the EVs proteome of *A. suum*. The actin 2 was also revealed in the EVs proteome of both *N. brasiliensis* and *B. malayi*. The ras-related protein (rab-11B) was recognized in the EVs proteome of *B. malayi*, while the heat shock protein 70 (HSP70) was also in common with *N. brasiliensis* (**Table 2**). No EVs proteins of *A. pegreffii* showed high similarity with EVs of *T. muris*.

**Table 2:** The top 10 *A. pegreffii* L3-EVs proteins identified in the EVs of *A. suum*, *N. brasiliensis* and *B. malayi*.

EVs from *A. pegreffii*	EVs from *A. suum*, *N. brasiliensis* and *B. malayi*			Blast Similarity (%)
Protein Name	UniProt Accession No.	Protein Name	Organism	
Actin 2	U1MSU7	Actin-2	*A. suum*	100
Actin 2	A0A0N4XTT2	Actin	*N. brasiliensis*	98.59
Actin 2	A0A0K0JJB8	BMA-ACT-5 GN=bma-act-5	*B. malayi*	96.94
Galectin	F1LAD2	Galectin	*A. suum*	94.94
Ras-related protein rab-11B	A0A0K0JE46	BMA-rab-11.1 GN=bma-rab-11.1	*B. malayi*	94.41
Adenylate kinase isoenzyme 1	F1LE77	Adenylate kinase isoenzyme 1	*A. suum*	94.20
Heat shock protein 70	A0A0N4XQZ6	Heat shock 70	*N. brasiliensis*	93.59
PPIase cyclophilin-type domain-containing protein	U1MF77	Triosephosphate isomerase	*A. suum*	93.50
Glyceraldehyde-3-phosphate dehydrogenase	A0A0M3K2Y3	Glyceraldehyde-3-phosphate dehydrogenase	*A. suum*	92.00
Propionyl-CoA carboxylase beta	KHN75145	Propionyl-carboxylase beta	*A. suum*	91.98

### 3.7 Identification of essential, potential pathogenicity-related and allergenic proteins

A total of 123 proteins were found to be “essential” for the survival of the parasite by DEG (Database of Essential Genes database). The top ten proteins with the highest similarity (ranging from 97 to 76,16%) against the proteins of this database are listed in **Table 3**. Similarly, 130 proteases/protease inhibitors were identified in the *A. pegreffii* EVs, by interpolating data with the peptidase of the MEROPS database. The top 10 proteins with the highest similarity (ranging from 100 to 98,91%), were listed in **Table 4**. Results were also blasted against the databases reporting potential pathogenic proteins, i.e. protVirDB (Protozoan Virulent Proteins Database), VICTORS (Virulence Factors Database) and (Virulence Factors of Pathogenic Bacteria). Four putative pathogenicity-related proteins with the highest similarity against these databases were identified. Results are reported in **Table 5**. Interestingly, the heat shock protein 70 was detected both in VICTORS and VIRDB databases. When the FARRP (Food Allergy Research and Resource Program) database was interrogated, a total of 20 proteins (**Table 6**) were identified as potential allergens. The AllerCatPro predicts that 14 proteins have a high possible known allergenic potential (**Table 6**).

**Table 3:** The top 10 matches of essential proteins detected in the EVs of *A. pegreffii* L3 by DEG database.

EVs from *A. pegreffii*	Essential proteins			Blast similarity (%)
Protein name	DEG accession no.	Protein name	Organism	
Actin-2	DEG20290735	Actin gamma 1	*Homo sapiens*	97.20
H(+)-transporting two-sector ATPase	DEG20020294	Vacuolar H ATPase family member (vha-13)	*Caenorhabditis elegans*	87.76
Heat shock protein 70	DEG20330753	Heat shock protein family A (HSP70) member 8	*Homo sapiens*	86.13
14-3-3 zeta	DEG20280061	14-3-3 zeta	*Bombyx mori*	85.77
Elongation factor 1-alpha	DEG20320075	Eukaryotic translation elongation factor 1 alpha 1	*Homo sapiens*	84.31
Adenosylhomocysteinase	DEG20020012	K02F2.2	*Caenorhabditis elegans*	81.70
Elongation factor 2	DEG20280147	Translation elongation factor 2	*Bombyx mori*	80.40
Heat shock protein 90	DEG20070168	HSP83	*Drosophila melanogaster*	78.23
Peroxiredoxin	DEG20201416	Peroxiredoxin 2	*Homo sapiens*	76.16
6-phosphogluconate dehydrogenase, decarboxylating	DEG20330069	Phosphogluconate dehydrogenase	*Homo sapiens*	71.07

**Table 4:** The top 10 matches of proteases/protease inhibitors detected in the EVs of *A. pegreffii* L3 by MEROPS database.

EVs from *A. pegreffii*	Protease/Protease Inhibitor				BLAST Similarity (%)
Protein name	MEROPS Accession No.	MEROPS classification	Activity	Organism	
Carboxypeptidase	MER1107341	family S10 non-peptidase homologues	Serine protease	*A. simplex*	100
Thyrotropin-releasing hormone-degrading ectoenzyme	MER1107292	family M1 unassigned peptidases	Metalloprotease	*A. simplex*	100
Gamma-glutamyltranspeptidase 1	MER1107372	family T3 unassigned peptidases	Threonine protease	*A. simplex*	99.80
M20_dimer domain-containing protein	MER1107182	carnosine dipeptidase II	Metalloprotease	*A. simplex*	99.79
M20_dimer domain-containing protein	MER1107377	carnosine dipeptidase II	Metalloprotease	*A. simplex*	99.78
M20_dimer domain-containing protein	MER1107451	carnosine dipeptidase II	Metalloprotease	*A. simplex*	99.36
Pepsin-I3 domain-containing protein	MER1107953	aspin	Aspartic protease inhibitor	*A. simplex*	99.28
CYTOSOL_AP domain-containing protein	MER1107977	family M17 non-peptidase homologues	Metalloprotease	*A. simplex*	99.06
Carboxypeptidase	MER1107913	family S10 non-peptidase homologues	Serine protease	*A. simplex*	98.95
Carboxylic ester hydrolase	MER1107193	family S9 non-peptidase homologues	Serine protease	*A. simplex*	98.91

**Table 5:** Top five putative pathogenicity-related proteins in EVs by protVirDB, VICTORS and VFDB.

EVs from *A. pegreffii*		Pathogenicity-related protein			Blast similarity (%)
Protein Name	Database	NCBI Accession No.	Protein name	Organism	
HSP70	protVirDB	AAF75871	HSP70, partial	*Cryptosporidium parvum*	78.96
	VICTORS	BAB20284	HSP70	*Toxoplasma gondii*	75.89
HSP 90	protVirDB	AAB97088	HSP90	*Eimeria tenella*	71.69
Peroxiredoxin	VICTORS	AAP68994	Thiol-specific antioxidant protein 1	*Cryptococcus neoformans* var. *grubii*	57.06
Glucose-6-phosphate isomerase	VFDB	WP_011272485	Glucose-6-phosphate isomerase	*Haemophilus influenzae*	52.01
Phosphoglucomutase 1	VICTORS	NP_697100	Phosphoglucomutase	*Brucella suis*	52.01

### 3.8 Predicted host-parasite protein interaction

**Figure 6:**
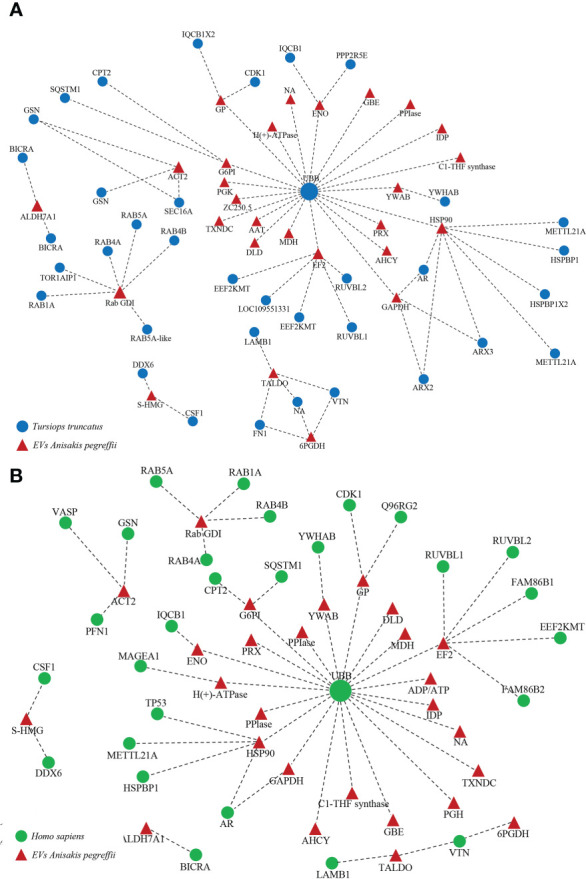
Cetacean **(A)** and human **(B)** host-parasite interaction network. Red triangles represent the *A. pegreffii* EVs proteins; blue circles represent the human proteins; green circles represent the cetacean proteins. The names of the proteins are given in the **Supplementary File 2**.

The interaction network among parasite proteins *versus* proteins of the definitive (cetaceans) and accidental (human) hosts was investigated. As prototype of a cetacean host, we have considered the bottlenose dolphin (*Tursiops truncatus*), which represents a natural definitive host of *A. pegreffii* across its range of distribution (Cipriani et al., 2022). When predicting the interaction between *A. pegreffii* and the bottlenose dolphin, a total of 27 EVs parasite proteins and 36 dolphin proteins were identified (**Figure 6A**; **Supplementary File 2**). L3-EV proteins that showed the highest number of potential interactions with the natural definitive host proteins were the heat shock protein 90 (HSP90) (8 interactions), the rab GDP dissociation inhibitor (rab GDI) (6 interactions), the elongation factor 2 (EF2) (6 interactions). Among the bottlenose dolphin’s proteins, the polyubiquitin-B isoform (UBB) showed potential interactions with the highest number of *Anisakis* proteins (20 interactions) (**Figure 6A**; **Supplementary File 2**).

**Table 6:** Potential allergens detected in the EVs of *A. pegreffii* L3.

EVs Proteins	FARRP Database Match				AllerCatPro
Protein name	NCBI Accession No.	Protein name	Organism	Blast Similarity (%)	Prediction
Ani s 14 allergen	BAT62430	Ani s 14 allergen	*A. simplex*	100	Strong evidence
Ani s 13 allergen	ASL68918	Ani s 13 allergen	*A. simplex*	99	Strong evidence
Ani s 1 allergen	AGC60035	Ani s 1 allergen	*A. simplex*	98	Strong evidence
PPIase cyclophilin-type domain-containing protein	AEB54655	Triosephosphate isomerase	*Procambarus clarkii*	97	Weak evidence
Peptidyl-prolyl cis-trans isomerase	AVV30163	Cyclophilin 0101	*Olea europaea*	93	Strong evidence
Heat shock protein 70	AOD75395	Heat shock-like protein	*Tyrophagus putrescentiae*	92	Strong evidence
Glyceraldehyde-3-phosphate dehydrogenase	XP_026782131	Glyceraldehyde-3-phosphate dehydrogenase	*Pangasianodon hypophthalmus*	91	Strong evidence
Glutathione S-transferase 1	P46436	Glutathione S- transferase 1	*A. suum*	90	Strong evidence
Peptidyl-prolyl cis-trans isomerase	AEY79726	Cyclophilin	*Daucus carota*	90	Strong evidence
2-phospho-D-glycerate hydro-lyase	ACH70931	Enolase 3-2	*Salmo salar*	88	Strong evidence
Glucose-6-phosphate isomerase	XP_026782721	Low quality protein: glucose-6-phosphate isomerase b	*Pangasianodon hypophthalmus*	88	Strong evidence
Fructose-bisphosphate aldolase	QBO59887	Pen c 1 allergen	*Penaeus chinensis*	87	Weak evidence
Fructose-bisphosphate aldolase	QBO59887	Pen c 1 allergen	*P. chinensis*	85	Strong evidence
Ferritin	AAG02250	Ferritin heavy chain-like protein	*Dermatophagoides pteronyssinus*	84	Weak evidence
Glycogen phosphorylase	CAA35238	Alpha/beta gliadin-like protein pro	*Triticum aestivum*	81	Strong evidence
Transaldolase	AHY02994	Transaldolase	*Fusarium proliferatum*	80	Weak evidence
Malate dehydrogenase	AF084828	Major allergenic protein Mal f4	*Malassezia furfur*	77	Weak evidence
Filamin-A	QFI57017	Filamin C	*Scylla paramamosai*	77	Strong evidence
Cytosolic 10-formyltetrahydrofolate dehydrogenase	AOD75396	Aldehyde dehydrogenase-like p	*Tyrophagus putrescentiae*	75	Weak evidence
UA3-recognized allergen	BAT62430	Ani s 14 allergen	*A. simplex*	75	Strong evidence

When predicting the interaction between *A. pegreffii* and the human host, a total of 27 EV parasite proteins and 29 human proteins were identified (**Figure 6B**; **Supplementary File 2**). The L3-EV proteins that showed the highest number of potential interactions with human proteins were the elongation factor 2 (EF2) (6 interactions), the heat shock protein 90 (HSP90) (5 interactions), the rab GDP dissociation inhibitor (rab GDI) (4 interactions), the actin (ACT2) (3 interactions). Among the human proteins, the polyubiquitin-C (UBB) showed potential interactions with the highest number of *Anisakis*’ proteins (21 interactions) (**Figure 6B**; **Supplementary File 2**).

## 4 Discussion

In recent years, a large effort has been devoted to investigate the molecular mechanisms evolved by the species of the *A. simplex* (s.l.) complex in order to colonise and survive in the host’s environment, as well as their ability to modulate the host immune response (Mehrdana and Buchmann, 2017; Palomba et al., 2020; Trumbić et al., 2021; Harbar et al., 2022). In this regard, besides the release of excreted/secreted products (ESPs), the shedding of EVs has been regarded as a powerful and plastic biological mechanism enabling nematodes, as well as other parasites to interact and successfully colonize the host microenvironment (Marcilla et al., 2014; Sánchez-López et al., 2021). So far, the proteomic profiling of ESPs has been described for the species *A. simplex* (s.s.) (Kochanowski et al., 2022), while no specific analysis is available for ESPs of *A. pegreffii*. Recently, EVs from the *A. pegreffii* L3 were shown to deliver miRNA (Cavallero et al., 2022a), while the proteomic information available for L3-EVs are referred to *Anisakis* sp. (Boysen et al., 2020), although the object of that study was likely the species *A. simplex* (s.s.) being the larvae collected from *Clupea harengus* of the NE Atlantic Ocean, which has been found parasitised by that species (Mattiucci et al., 2018b). In the present study, the protein repertoire secreted by the genetically identified *A. pegreffii* L3, in particular that associated with shed EVs, has been characterised.

NTA and TEM analyses, carried out in this study, allowed the characterization of prevalent 80-260 nm vesicles subset, a range size including both exosome-like, likely derived from endocytic pathways and microvescicles-ectosomes, possibly originated from the shedding of the cytoplasmic membrane (Eichenberger et al., 2018c; Mazanec et al., 2021).

The proteomic profiling allowed the characterization of 153 proteins contained in the secretome of *A. pegreffii*, in particular the one associated with L3-EVs. The L3-EVs had a high P/μg ratio, which has been proposed as a good approach to evaluate EV purity (Webber and Clayton, 2013; Théry et al., 2018), although co-precipitation of soluble proteins with EVs could not definitely be excluded.

Among the 153 identified proteins, associated to L3-EVs of *A. pegreffii*, a high correspondence was found with 11 proteins observed also in the EVs proteomic repertoire of *Anisakis* spp. provided by Boysen et al. (2020) i.e., the chloride intracellular channel exc-4, the actin 2, the pepsin-I3 domain-containing protein, the p-type domain-containing protein, glutamate dehydrogenase (NAD(P)(+)), heat shock protein 70, 14-3-3 zeta, ras-related protein Rab-11B and three unnamed proteins (**Table 1**). Among the proteins detected by Boysen et al. (2020), the tubulin beta, the ATP synthase F1 (alpha + beta subunit), RAS-like GTP-binding protein RhoA, the superoxide dismutase, and the ADP ribosylation factor 1, were not identified in the present EVs database.

Additionally, the EVs proteomic composition of *A. pegreffii* here provided, showed similarity with the actin, ras-related protein, adenylate kinase, triosephosphate isomerase, the galectin, and the heat shock protein of *A. suum*, *N. brasiliensis* and *B. malayi*. Indeed, actin is the most abundant cytoskeletal protein contained in EVs of other nematode parasites and is considered an EV marker (Sotillo et al., 2020). Its role is mainly related to cell division and motility, muscle contraction and other cellular processes. The presence of actin in the *A. pegreffii* L3-EVs would be related to its life-history stage. The thermal condition (37°C) used in this study represents a physical cue stimulus able to enhance the capacity of L3 to moult into L4 as reported by *in vitro* studies of parasites of the *A. simplex* (s. l.) complex (Mattiucci et al., 1986; Iglesias et al., 2001). Adenylate kinase (AK) and triosephosphate isomerase (TPI) are enzymes involved in metabolic regulation and well-conserved proteins among species (Knowles, 1991). It is intriguing to note that, both enzymes have also been associated with nematode infection and described as potential immunomodulators. TPI secretion by *B. malayi* microfilaria sustains the development and contributes to altering the host T helper (Th) cell balance (Hewitson et al., 2014); while AK was shown to be a potential target for preventing *Schistosoma japonicum* infection (Gao et al., 2017). The possible contribution of L3-EVs in modulating the microenvironment by altering the immune surveillance of the host tissues is also strongly suggested by the identification of immune-modulating molecules HSPs and galectin as protein components carried by L3-EVs.

HSPs are synthesized in response to cellular stress and are a common mechanism among organisms. They stabilize conformational assembly of newly synthetized polypeptides and allow degradation of the unfolded ones (Pérez-Morales and Espinoza, 2015). HSPs have been found as common component of nematode EVs and regarded as EV marker (Eichenberger et al., 2018a; Eichenberger et al., 2018b). In particular, HSP70 associated with *Anisakis* EVs, identified in this study, has been found also as EV protein content by Boysen et al. (2020). The presence of HSPs in the L3-EVs seems to support their role in mediating and/or buffering the thermal and osmotic stress that the parasite undergoes to during its life cycle, passing from the marine environment to the homeothermic natural/accidental host. HSPs also play a key role in the activation of innate immunity, acting as alarmins and inducing maturation of the antigen presenting cells (APCs) and providing polypeptides for triggering adaptive immune responses. HSPs released during nematode infection may contribute to increase the inflammatory microenvironment and be exploited by the pathogen to skew the host immune response (Hansen et al., 2019).

Other identified proteins i.e., 14-3-3 protein, annexin, galectin, GST, peroxiredoxin, SCP and thioredoxin, have been already reported in several EVs of helminth species and also retained having potential effects on the host immune system (Eichenberger et al., 2018c).

Among the top 10 A*. pegreffii* L3-EVs proteins identified as associated with the EVs of *A. suum*, *N. brasiliensis* and *B. malayi*, a high percentage of similarity (i.e., 94.94%) has been demonstrated for galectin of *A. suum* (**Table 1**). Galectins are lectins that bind N-acetyllactosamine-containing glycans, either in the free form or as components of glycoproteins or glycolipids. They play an immunoregulatory role in homeostasis as well as in disease (Thiemann and Baum, 2016). Nematode galectins have a similar structure to the human, although are not an ortholog of the mammalian galectins (Houzelstein et al., 2004) and it has been speculated that they have evolved to mimic host galectin (Tang et al., 2014). They seem to play a crucial role in defusing the host immune response by several mechanisms such as binding to host mucosal cells to induce a Th2 cytokine-promoting microenvironment (Xu et al., 2018). For instance, galectins produced by the terrestrial ascarid *Toxascaris leonina*, limit the host inflammatory response by inhibiting Th1 and Th2 cytokines production (Kim et al., 2010), while galectin-1 from *Angiostronlgylus cantonensis* induces apoptosis of macrophages (Shi et al., 2020). A potent mechanism that nematode galectins may exert is to hijack heavily glycosylated IgE. In fact, they also bind the carbohydrate moieties that decorate the IgE (Klion and Donelson, 1994). By this strategy, once the nematode galectins trap the IgE, they likely alter the IgE binding to mast cells and histamine degranulation, dampening the inflammatory triggering. To our knowledge, this is the first report describing the presence of galectin in L3-EVs of *A. pegreffii*. Further studies are required to understand the role played by *A. pegreffii* galectin during the parasite invasion.

A major component of the proteomic repertoire found associated to EVs released by *A. pegreffii* larvae is represented by proteases, mainly belonging to hydrolases, transferases, and reductases classes with pivotal roles in mammalian host-parasite interactions. In general, proteases have been reported to be abundant in EVs of helminth parasites (Eichenberger et al., 2018c; Hansen et al., 2019) and proposed to be involved in the parasite’s metabolic food processing and host immunomodulation. Among hydrolases, the metallopeptidases, the aspartic proteases and the serine proteases can play an important role in host-parasite interactions. Metallopeptidases are involved in the invasion of host tissues by the parasite, as they can degrade the extracellular matrix, and are also involved in the process of ecdysis and digestion of nutrients (Malagón et al., 2013). High levels of transcripts of a metallopeptidase (*nas*10) were found in the L3 infecting the muscle of the host fish *Micromesistius potassou* (Palomba et al., 2020). Aspartic proteases are actively involved in the growth and protection against host immune response and parasite’ moult, as well as they have been identified as virulence factors (Malagón et al., 2013; Trumbić et al., 2021). Serine proteases of *A. simplex* have been postulated to be related to the visceral migration in host tissues. The association of serine proteases to the L3-EVs of *A. pegreffii* seems to support the hypothesis that homeothermic temperature may be a stimulus for the synthesis and release of proteins that could modify the tissue microenvironment of the host to allow successful nematode infection. It is interesting to note that a Kunitz-type serine protease inhibitor has also been detected as EV component. In this case, the protease inhibitors from L3-EVs may inhibit the host proteases encountered in the digestive system or during larval migration (Palomba et al., 2020). This, in turn, would serve also to prevent or limit the host tissue damage due to the larval migration (Morris and Sakanari, 1994). Discovering the basis of the human host tissue penetration in the case of L3 in the gastrointestinal tract will be an important step in understanding the pathogenesis of the disease and further studies are warranted.

The characterization of the interactome is crucial to underpin host-parasite interactions. It is interesting to note that when performing network analysis of the predicted proteins interactions between *A. pegreffii* and its natural definitive (dolphin) or the accidental (human) host, both ubiquitin proteins from the two mammal hosts were the main protein interacting with most of the L3-EV proteins. In particular, human parasite interactions have shown that human ubiquitin is predicted to interact with the highest number of proteins. The ubiquitination process is crucial for proteasome-mediated protein degradation and relevant for the antigen presentation pathways. The protein-protein interaction pattern here detected is suggestive of a possible role of host ubiquitin as a defence strategy to dismantle the parasite proteins at cytoplasmic levels and possibly generate a parasite peptide repertoire available for antigen presentation to the host immune system. Despite this being only speculation, indeed proteomic profiling revealed that the L3-EVs associated proteins matched with the allergens as identified in several databases. Anis1, Anis14, and Anis13 are among those reported by the allergome database and registered as *Anisakis* allergenic proteins by WHO/IUIS. All of them are molecules with specific biological functions i.e., Anis1 is a Kunitz-type serine protease inhibitor and Anis13 is the myoglobin. Actually, Anis14 has an unknown biological role.

Interestingly, Anis14 was putatively retained to be a somatic/secreted allergen; this study seems to support the prediction that it is a secreted enzyme. Analogously, Anis13 (myoglobin), one of the major antigens of *A. pegreffii*, genetically characterised (Mattiucci et al., 2017), is identified as a target of IgE immune response during the human infection by the L3 stage of *A. pegreffii* (Mattiucci et al., 2017). So far, the role of Anis13*/*myglobin is not yet completely known. It was supposed that the myoglobin of *A. simplex* (s.s.) increases its protection against the host immune response (Palomba et al., 2020). However, at the temperature of the homothermic host, high transcript levels of the haemoglobin coding gene were detected (Palomba et al., 2019). This evidence is suggestive of a possible role of Anis13/myoglobin in the adaptation to the natural host, and in the pathogenic mechanisms of invasion in the accidental one. By using the FARRP database, several other putative predicted allergens were catalogued in *A. pegreffii* L3-EVs. In particular, the fructose-diphosphate-aldolase, transaldolase, and triosephosphate isomerase detected in L3-EVs were found similar in the proteomic profiling of the secretome of *A. simplex* (Kochanowski et al., 2022). Interestingly, a similar pathway of interactome was recently observed in the secretome analysis of *A. simplex* (Kochanowski et al., 2022). This finding supports the concept that proteins present in the secretome of the parasite species are released by EVs.

## 5 Conclusions

This study provides further knowledge on the characterisation of proteomic repertoire associated with L3-EVs released by *A. pegreffii* under the condition of 37°C. Most of the characterized proteins may play a role in the interaction with homeothermic definitive (marine mammals) and accidental (humans) hosts, suggesting that secretome associated to L3-EVs, we have described, might help the larva to penetrate the tissue host and possibly interfere with the host immune response as a “survival” mechanism allowing the parasite to moult to the fourth stage and to remain in that suitable niche even for a long time. At the same time, the release of such protein repertoire (i.e. proteinase inhibitors, HSPs, and some known allergens) may trigger pathogenic reaction in an accidental host which is at the base of the zoonotic disease, i.e. anisakiasis. However, because the different culture conditions *in vitro* used by us and several authors (DMEM, RPMI, M9 with or without different addictive) (i.e., Iglesias et al., 2001; Boysen et al., 2020; Duguet et al., 2020; Mazanec et al., 2021; Cavallero et al., 2022b) are a quite distant systems from the physiological environment in which the larva grows and develops or exerts its pathogenic role, gastrointestinal organoids would offer the closest experimental setting further to study nematode development and interaction with host microenvironment. In this regard, organoids have also been proposed to study the effect of nematode excretory/secretory products on host tissue microenvironment (White et al., 2022b). This experimental model may allow to study the biological influence of the “corona” whose protein composition is modulated by the tissue microenvironment and contribute to the surface interactome of EVs (Tóth et al., 2021).

Overall, the obtained EVs proteomic repertoire here described and discussed can provide a useful baseline for future comparative analyses, in order to understand the biology and the evolutionary adaptation of *A. pegreffii* to heterothermic and homeothermic hosts, as well as the mechanism of pathogenesis in those accidental ones.

## Data availability statement

The datasets presented in this study can be found in online repositories. The names of the repository/repositories and accession number(s) can be found in the article/**Supplementary Material**.

## Author contributions

Conceptualization; MP, AR, SM. Formal analysis: MP, AR, GM, MES, SM. Methodology: MP, AR, GM, TC, HR, CN, LM, VP, AG, MS, SM. Resources: MP, MS, SM. Writing – original draft: MP, AR, SM. Writing – review and editing: MP, GM, TC, HR, CN, LM, AG, MS, MES, BM, SM. All authors contributed to the article and approved the submitted version.
