# Supplementary figures and images for: Proteomic characterization of extracellular vesicles released by third stage larvae of the zoonotic parasite Anisakis pegreffii (Nematoda: Anisakidae)
Source: Front Cell Infect Microbiol. 2023 Mar 15;13:1079991. doi: 10.3389/fcimb.2023.1079991 (PMC10050594; doi:10.3389/fcimb.2023.1079991)

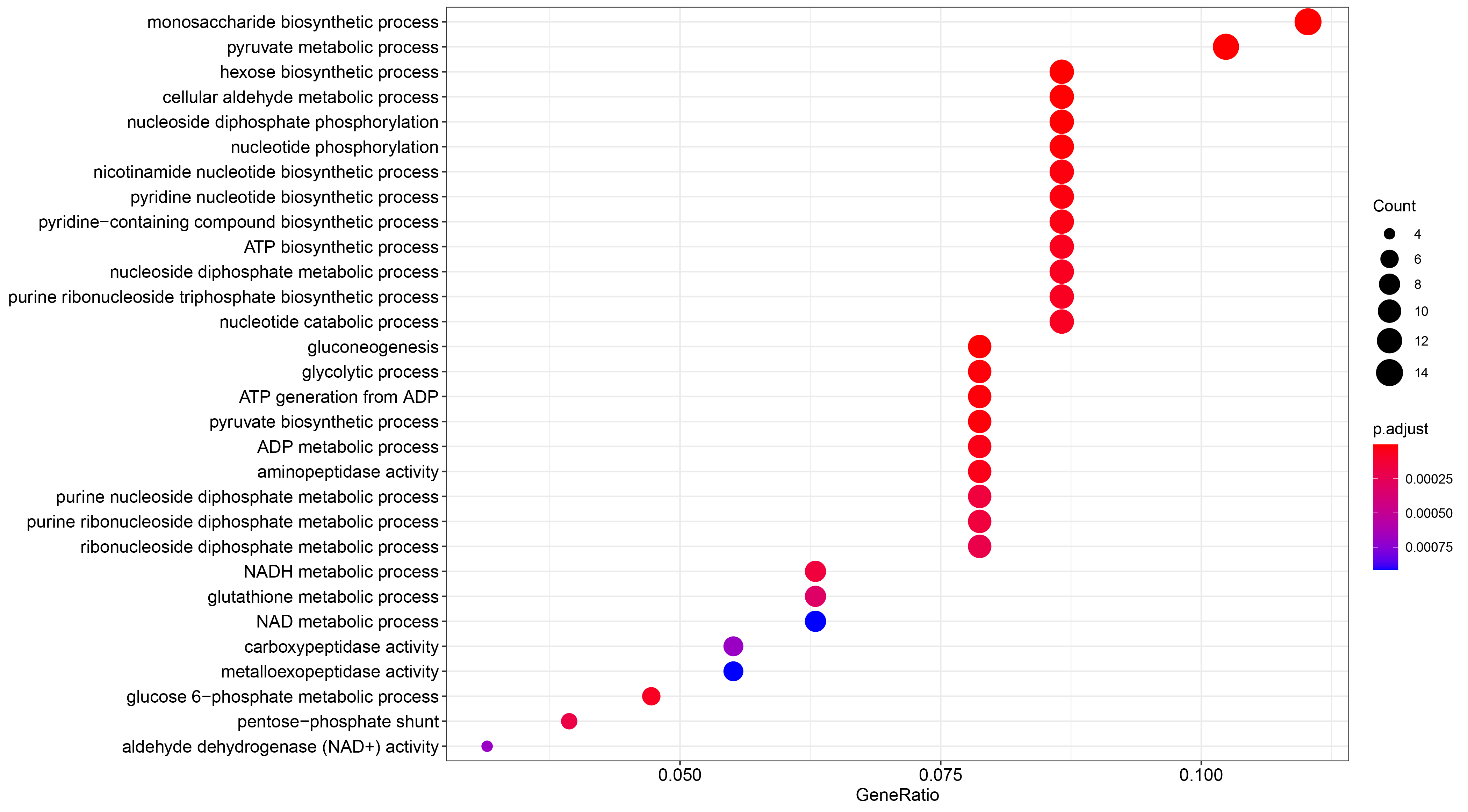

Supplement: Supplementary file 1 [file Image_1.jpeg]

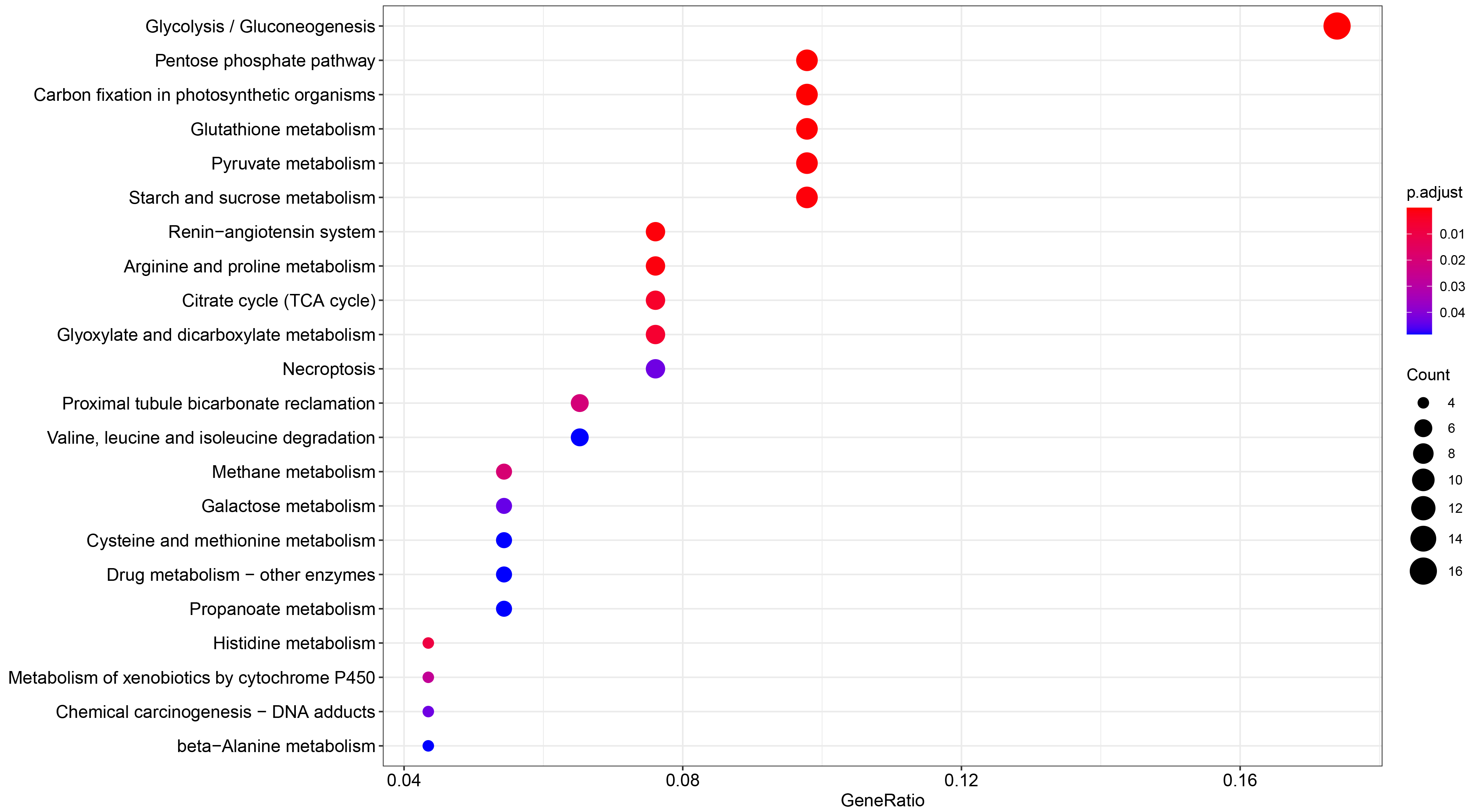

Supplement: Supplementary file 2 [file Image_2.jpeg]
