# Supplementary material for: Proteomic characterization of extracellular vesicles released by third stage larvae of the zoonotic parasite Anisakis pegreffii (Nematoda: Anisakidae)
Source: Front Cell Infect Microbiol. 2023 Mar 15;13:1079991. doi: 10.3389/fcimb.2023.1079991 (PMC10050594; doi:10.3389/fcimb.2023.1079991)
Supplement: Supplementary file 5 [file Table_3.docx]

Supplementary Material

Extracellular vesicles from the third-stage larvae of the zoonotic parasite *Anisakis pegreffii* deliver proteins implicated in host-parasite adaptation and pathogenesis

**Marialetizia Palomba^1¶^, Aurelia Rughetti^2¶^, Giuseppina Mignogna^3^, Tiziana Castrignanò^1^, Hassan Rahimi^2^, Laura Masuelli^2^, Chiara Napoletano^2^, Valentina Pinna^1^, Alessandra Giorgi^3^, Mario Santoro^4^, Maria Eugenia Schinina^3^, Bruno Maras^3^, Simonetta Mattiucci^5*^**

*** Correspondence:** Corresponding Author: [simonetta.mattiucci@uniroma1.it](mailto:simonetta.mattiucci@uniroma1.it)

**Supplementary Figure 1.** Analysis of enriched gene ontology (GO) terms. The size of each dot represents the number of proteins enriched in that term, while the color represents the corrected p-value. p.adjust<0.05 was considered significant.

**Supplementary Figure 2.** Analysis of enriched KEGG pathway. The size of each dot represents the number of proteins enriched in that pathway, while the color represents the corrected p-value. p.adjust<0.05 was considered significant.

**Supplementary Data 1.** List of proteins identified by MS from EVs isolated from *A. pegreffii* L3.

**Supplementary Data 2.** S2 File. List of codes reported in Figure 6.
